# Supplementary material for: Coupling optogenetics and light-sheet microscopy, a method to study Wnt signaling during embryogenesis
Source: Sci Rep. 2017 Nov 30;7:16636. doi: 10.1038/s41598-017-16879-0 (PMC5709371; doi:10.1038/s41598-017-16879-0)

Coupling optogenetics and light-sheet microscopy, a method to study Wnt signaling during embryogenesis

Prameet Kaur, Timothy E. Saunders, Nicholas S. Tolwinski

Supplementary Videos Legends

Supplementary Video 1. Arm-CRY2-mCh puncta formation in *Drosophila* embryos overexpressing Arm-CRY2-mCh in a wild-type background upon exposure to 488nm laser light. The whole embryo was illuminated with 488nm laser light at 2.5 minute intervals for half an hour to activate oligomerization. Puncta formation observed during illumination was reversible and oligomerization could be induced repeatedly.

Supplementary Video 2A. Arm-CRY2-mCh overexpressing arm^{XM19} mutant embryos not exposed to light showed diffuse localization of Arm-CRY2-mCh and normal embryonic development.

Supplementary Video 2B. Arm-CRY2-mCh overexpressing arm^{XM19} mutant embryos exposed to 488nm light showed distinct puncta formation of Arm-CRY2-mCh and various developmental defects as compared to embryos not exposed to light.

Suppl**ementary Video 2C.** Embryos expressing Arm-EGFP showed normal embryonic development.

Supplementary Video 3A. Spatial perturbation of Wnt signaling in Arm-CRY2-mCh overexpressing *arm*^{XM19} mutant embryos. The right half of the embryo was illuminated with 488nm laser light and the whole embryo was imaged simultaneously in the red channel. The right side of the embryo (with blue light illumination) showed distinct clustering and developmental defects.

Supplementary Video 3B. Spatial perturbation of Wnt signaling in Arm-CRY2-mCh overexpressing *arm*^{XM19} mutant embryos. The posterior region of the embryo was illuminated with 488nm laser light and the whole embryo was imaged simultaneously in the red channel. The posterior region of the embryo (with blue light illumination) showed distinct clustering and incomplete germ band retraction.

Supplementary Figure 1A. Armadillo-Cry2-mCherry fusion constructs. Fusion constructs were generated using the MultiSite Gateway® Pro 2.0 cloning kit (Invitrogen).

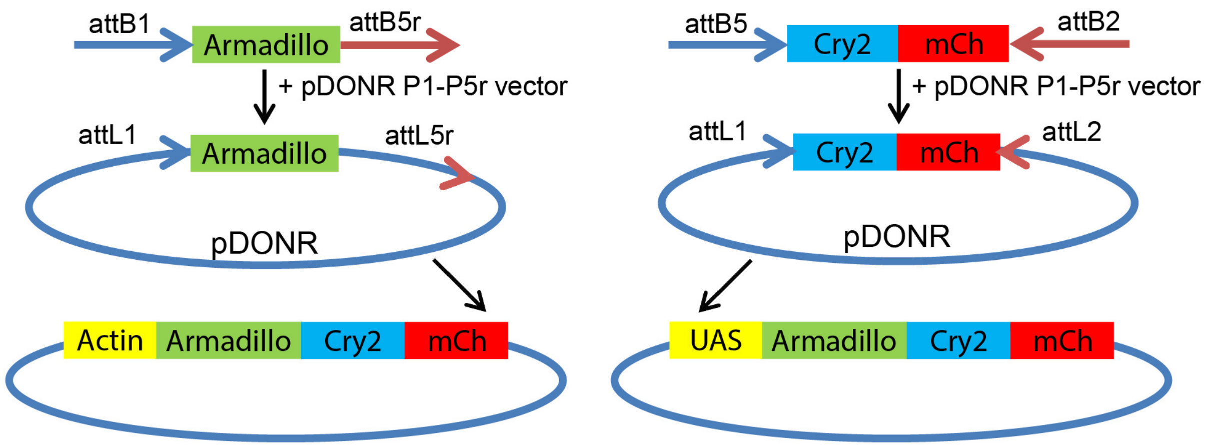

Supplement: Supplementary file 1 — Supplementary Information [file 41598_2017_16879_MOESM1_ESM.pdf]
